# Molecular diagnosis of *Pseudoterranova decipiens* s.s in human, France

**DOI:** 10.1186/s12879-017-2493-7

**Published:** 2017-06-06

**Authors:** Julie Brunet, Bernard Pesson, Maude Royant, Jean-Philippe Lemoine, Alexander W. Pfaff, Ahmed Abou-Bacar, Hélène Yera, Emilie Fréalle, Jean Dupouy-Camet, Gema Merino-Espinosa, Magdalena Gómez-Mateos, Joaquina Martin-Sanchez, Ermanno Candolfi

**Affiliations:** 1 Laboratoire de Parasitologie et de Mycologie Médicale, Plateau Technique de Microbiologie, Hôpitaux Universitaires de Strasbourg, 1 place de l’Hôpital, BP 426, F-67091 Strasbourg cedex, France; 2 Institut de Parasitologie et Pathologie Tropicale, EA 7292, Fédération de Médecine Translationnelle, Université de Strasbourg, 3 rue Koeberlé, F-67000 Strasbourg, France; 3 Service des consultations externes, Hôpitaux Universitaires de Strasbourg, 1 place de l’Hôpital, BP 426, F-67091 Strasbourg cedex, France; 4 Service de Parasitologie-Mycologie, Hôpital Cochin, Hôpitaux Universitaires Paris Centre, Assistance Publique Hôpitaux de Paris, Université Paris Descartes, 27 rue du Faubourg St Jacques, F-75015 Paris, France; 5 CHU Lille, Laboratoire de Parasitologie-Mycologie et Université de Lille, CNRS, Inserm, Institut Pasteur de Lille, U1019 – UMR 8204 – CIIL – Center for Infection and Immunity, Lille, France; 6 Departamento de Parasitología, Facultad de Farmacia, Universidad de Granada, Campus Universitario de Cartuja s, /n 18071 Granada, Spain

**Keywords:** *Pseudoterranova decipiens*, Anisakidae, Nematode, Molecular identification, Human infection, France

## Abstract

**Background:**

*Anisakis* and *Pseudoterranova* are the main genera involved in human infections caused by nematodes of the Anisakidae family. Species identification is complicated due to the lack of differential morphological characteristics at the larval stage, thus requiring molecular differentiation. *Pseudoterranova* larvae ingested through raw fish are spontaneously eliminated in most cases, but mechanical removal by means of endoscopy might be required. To date, only very few cases of *Pseudoterranova* infection have been reported in France.

**Case presentation:**

A 19-year-old woman from Northeastern France detected, while brushing her teeth, a larva exiting through her mouth. The patient who presented with headache, diarrhea, and abdominal cramps reported having eaten baked cod. The worm was a fourth-stage larva with a size of 22 × 0.9 mm, and molecular biology identified it as *Pseudoterranova decipiens* sensu stricto (s. s.). In a second *P. decipiens* infection case, occurring a few months later, a worm exited through the patient’s nose after she had eaten raw sea bream.

**Conclusion:**

These two cases demonstrate that *Pseudoterranova* infection is not uncommon among French patients. Therefore, molecular techniques should be more widely applied for a better characterization of anisakidosis epidemiology in France.

## Background

Several helminth parasites of fish can infect humans, the most common genus being *Anisakis*, usually *A. simplex* s.s and *A. pegreffii* and less commonly *Pseudoterranova* and *Contracaecum* genera. Anisakidosis is encountered worldwide, though its prevalence is higher in countries where fish is primarily consumed raw. About 20,000 cases of anisakidosis have been reported worldwide, most of them in Japan [1–3]. *Pseudoterranova decipiens (P. decipiens*) is the second most common *Anisakidae* found in humans [1, 4]*.* While rare in Europe, this nematode species is more frequently found in North America, Japan, Korea, and Chile [5–9]. A number of sibling species belong to this *P. decipiens* complex that is genetically, though not morphologically, distinguishable [10].

Adult of *P. decipiens* are mainly found in the gastrointestinal tract of pinnipeds (seals, sea lions, and walruses) that serve as definitive hosts [1, 4], while planktonic or benthic crustaceans act as intermediate hosts. Several fish species (natural second intermediate paratenic hosts) can be infected by third-stage larvae of *P. decipiens,* particularly Pacific cod, red snapper, hake, and Pacific halibut. A *P. decipiens* prevalence of up to 55% in Baltic cod was reported in the Southern Baltic Sea [1, 9, 11]. Encapsulated larvae reside mainly in visceral organs and peritoneal cavity of the fish, but some may migrate into the musculature [2].

Human infection is the result of accidental ingestion of the third-stage larvae present in viscerae or muscles of a wide range of marine fish or squids. Raw, undercooked, or marinated fish is a source of human contamination. In humans, the larvae do not develop into adult worms [1–4].

From 2010 to 2014, 19 cases of anisakidosis were reported in France, but this figure is probably underestimated in view of the parasite’s high prevalence in fish [12]. The incidence of pseudoterranovosis in France has not yet been properly assessed as species diagnosis is rarely made, yet at least two cases have been reported following cod ceviche and raw coalfish consumption [13, 14].

Although humans are accidental hosts for this nematode parasite in which they cannot complete its life cycle, *P. decipiens* may cause serious disease. Symptoms usually appear 1-7 h after ingestion [4]. Then, the infection tends to be mostly asymptomatic, and larvae are usually coughed up by patients 36 h to 7 days following ingestion. Associated nausea and foreign body sensation between the teeth were also reported [1, 5, 8, 15, 16].

Rare cases of invasive pseudoterranovosis may occur in humans. Larvae mostly penetrate the gastric mucosa, commonly causing severe epigastric pain. Larvae are extracted via endoscopy, resulting in rapid pain relief [7, 14, 17].

We have reported herein two cases of pseudoterranovosis that molecular biology identified as *P. decipiens* s.s.

## Case presentation

A 19-year-old woman living in Strasbourg (Northeastern France) complained of headache, diarrhea, asthenia, anorexia, and abdominal cramps having lasted for 15 days. The symptoms started after she had consumed baked cod. Two nematode larvae were spontaneously expelled via the mouth while she was brushing her teeth. The patient had an unremarkable medical history, and had not recently traveled abroad. The patient’s discomfort improved after worm expulsion, and gastroscopy proved negative. The patient was treated with albendazole 800 mg/day for 5 days.

Stool examination and blood analysis were all normal without eosinophilia. Serology was negative for *Trichinella spiralis*, *Strongyloides stercoralis*, *Ascaris lumbricoides,* and *Toxocara canis*.

*Anisakis* serology was also negative. IgG titers against *Anisakis sp.* were estimated at 1:50 (<1:100) using an own laboratory-developed Immunofluorescence assay (Parasitology-Mycology laboratory, Hôpital Cochin). Detection of anti-*Anisakis* antibodies using immunoelectrophoresis was negative and IgE titres against *Anisakis* sp*.* (p4) were at <0.1kUa using ImmunoCAP (Phadia, France) (Parasitology-Mycology laboratory, Lille University hospital center). No IgE against cod were detected by means of ImmunoCAP.

**Fig. 1:**
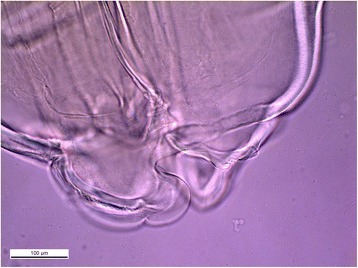
*P. decipiens* sensu stricto anterior region with three anterior lips without any boring tooth

**Fig. 2:**
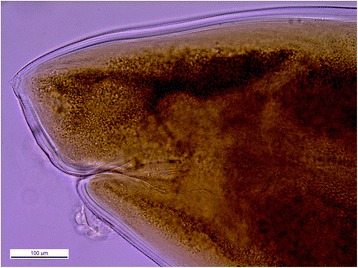
*P. decipiens* sensu stricto tail with mucron

The larva was brought to the Parasitology department of Strasbourg University Hospital for further characterization. The parasite was 22 mm long and 0.9 mm large with three anterior lips without any boring tooth (Figure 1). Morphological characteristics were a clearly visible esophagus and ventriculus, with a terminal tiny point (Figure 2). The parasite was morphologically characterized as a fourth-stage *Anisakidae* larva and submitted for molecular species identification at the department of Parasitology, University of Granada, Spain.

DNA was extracted from a roundworm portion using the commercial kit RealPure kit for genomic DNA extraction (REAL), following mechanical rupture of parasite tissue using a pestle and repeated freezing/thawing cycles in liquid N_2_. The precipitated pellet was resuspended in 20 μL of deionized water. For genetic identification of the larva, polymerase chain reaction of the ribosomal fragment ITS1–5,8S–ITS2 was carried out with primers NC5 (forward): 5′ GTAGGTGAACCTGCGGAAGGATCATT 3′ and NC2 (reverse) 5′ TTAGTTTCTTTTCCTCCGCT 3′ using programming described in 1998 by Zhu et al. [18]. The expected size of the amplified fragment was approximately 1000 bp. As controls, two specimens previously identified by the same technique as *A. pegreffii* and *A. simplex* s.s., were used respectively [19]. PCR products were run on gels prior to digestion in order to verify the amplification process’s success.

First, restriction fragment length polymorphism (RFLP) was performed independently with two restriction enzymes, *TaqI* (5′...T↓CGA...3′) and *HinfI* (5′...G↓ANT...3′) Fast Digest (Thermo Sciencific) at 65 °C and 37 °C, respectively for 10 min, using a final enzyme concentration of 0.5 U/μL. The results were visualized through electrophoresis on a 3% agarose gel. The band pattern collected from the roundworm differed from *A. simplex* s.s. and *A. pegreffii* controls.

**Fig. 3:**
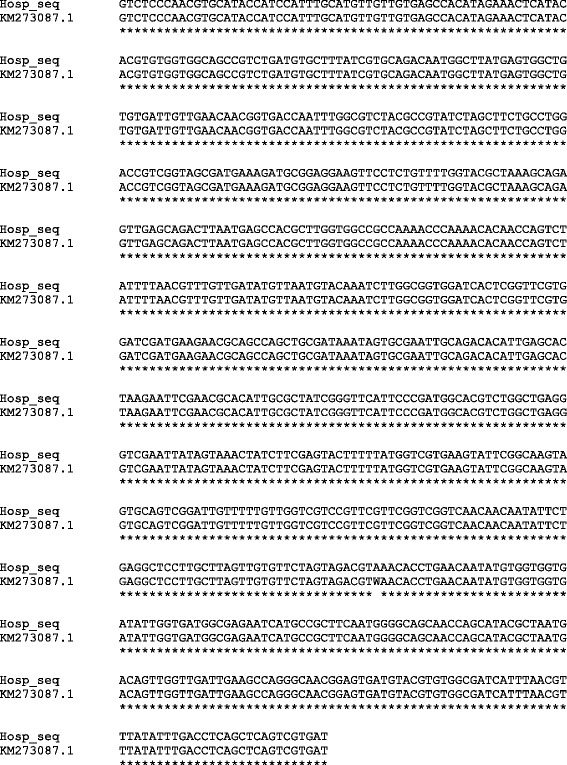
Sequence alignment detailed of *P. decipiens* sensu stricto of this case

For comparative sequence analysis, the PCR amplification product was purified using the Zymoclean Gel DNA Recovery Kit (Zymo Research), and then directly sequenced in both directions using the same primers as for DNA amplification. The sequences were edited and aligned using Clustal-X 1.81 software. Subsequently, BLAST software was employed to compare this sequence with a sequence library in order to identify similar sequences. As shown in Fig. 3, two identical sequences of *P. decipiens* s.s. from Denmark (GenBank accession number KM273087.1) and Norway (GenBank accession number JQ673262.1) showed a 99% similarity with our sequence. The alignment has been detailed in Fig. 3.

In a second infection case occurring a few months later, a worm exited through the patient’s nose after she had eaten raw sea bream. The larva was morphological and molecularly identical to the previous case being identified as *P. decipiens* s.s.

## Conclusion

Incidence of *Pseudoterranova* infection in France has not yet been assessed; owing to the lack of specific symptoms, the condition is most likely underdiagnosed. In most of the described cases, infections refer to *Pseudoterranova* sp., whereas at least eight sibling species of *Pseudoterranova* were genetically differentiated with distinct geographical and host ranges: *P. decipiens s.s.*, *P. kogiae*, *P. ceticola*, *P. decipiens E*, *P. cattani*, *P. azarasi*, *P. krabbei, and P. bulbosa,* which are morphologically indistinguishable [20]. In our cases, molecular biology allowed us to precisely identify the fourth-stage larvae as *P. decipiens* s.s.

*P. decipiens* s.s. is principally located in the Arctic and subarctic regions. Adult stages are frequently found in common seal, grey seal, and *Phocidae*. Larval forms are mostly encountered in *Gadidae* fish species like coalfish or cod [10].

There is a distinct difference in the clinical syndromes of human pseudoterranoviasis between those encountered in Japan and the USA. In Japan, the disease is rather severe, whereas in the USA, the condition is milder and without clear symptoms attributable to gastro-intestinal invasion [21]. Whether the clinical manifestations differ among *P. decipiens* species are still to be determined, partly due to only tentative species identification in most reported cases. However, *P. decipiens* larvae seem to be less invasive than those of the *Anisakis simplex* complex [1, 15]. Absent severe gastric symptoms were reported in *P. azarasi* and *P. cattani* infections [15, 16]. For *P. azarasi* infection, which applied to our patient, morphological diagnosis was made based on orally expelled fourth-stage larvae, associated to the lack of a boring tooth [16]. Third- or fourth-stage *Pseudoterranova* larvae can cause human infections [5]. Third-stage larvae invade gastric mucosa where they molt to fourth-stage larvae, without ever reaching the adult stage in humans [5, 14, 16]. For *A. simplex* in humans, the parasite passes from third- to fourth-stage larva within 3 to 4 days following ingestion [22]. To date, only two cases of human infection caused by *P. decipiens* larvae have been reported in France. In the first case, reported in 1996, a 40-year-old woman presented with epigastric pain. A larva was removed from the epigastric mucosa via gastroscopy, and morphologically identified as fourth-stage larva of *P. decipiens* sp. The second case, reported in 2014, concerned a 25-year-old woman. A larva was expectorated by the patient and identified as *Pseudoterranova* sp. [13, 14].

Clinical diagnosis proves to be difficult due to non-specific symptoms. Diagnosis is primarily made when the patient coughs up or vomits the worm. Endoscopic examination may be useful, though gastric *pseudoterranovosis* is commonly misdiagnosed as a peptic ulcer. Interpretation of immunological tests is often difficult owing to cross-reactivity with antigens from closely related nematode species like *Ascaris* or *Toxocara* [1, 4]. In the rare event of *Pseudoterranova* infection detected by *Anisakis* serology, the test was negative for IgG (1:50 using own laboratory-developed IFA) yet positive for IgE (1.4kUa ImmunoCAP) [13]. In this case, and contrary to our cases, the patient suffered from symptoms a few hours after having consumed contaminated raw fish.

Molecular biology analysis, while rarely carried out, has nevertheless epidemiological usefulness to determine the specific species within the genus. Since 2015, two cases of anisakidosis have been diagnosed at Strasbourg University Hospital, with *P. decipiens* identified in both cases. In the second case, the patient spontaneously expelled one larva through the nose after having eaten raw sea bream.

Extraction is the preferred treatment if the larva is not spontaneously coughed up. In most cases, abdominal discomfort gradually decreases following worm removal. Several studies suggest that albendazole (400-800 mg/day for 6–21 days) may result effective [1, 23].

No specific pharmacological treatment exists to effectively kill live larvae in seafood, and the best protection consists of individual prevention. The infection risk is clearly related to traditions of consuming raw, lightly cooked, or marinated fish (sushi, gravlax, ceviche, etc.), even more so if the fish is eaten whole. The contamination risk can be reduced by appropriate preparation of fish and squid. Larvae are killed by temperatures higher than 60 °C over at least 1 min, freezing at −20 °C for 7 days, or freezing at −35 °C for at least 15 h [1–4].

## Abbreviations

RFLP: restriction fragment length polymorphism

## References

[1] Hochberg NS, Hamer DH (2010). Anisakidosis: perils of the deep. Clin Infect Dis.

[2] Fréalle E, Gay M, Touabet N, Seesao Y, Dutoit E, Yera H, Certad G, Dupouy-Camet J, Viscogliosi E, Aliouat-Denis CM. L’anisakidose, une helminthose humaine aux manifestations allergiques émergentes. Feuillets de biologie.2016; 328:27–38.

[3] Shamsi S, Butcher AR (2011). First report of human anisakidosis in Australia. Med J Aust.

[4] Chai JY, Darwin Murrell K, Lymbery AJ (2005). Fish-borne parasitic zoonoses: status and issues. Int J Parasitol.

[5] Mercado R, Torres P, Munoz V, Apt W (2001). Human infection by *Pseudoterranova decipiens* (Nematoda, Anisakidae) in Chile: report of seven cases. Mem Inst Oswaldo Cruz.

[6] Yu JR, Seo M, Kim YW, Oh MH, Sohn WM (2001). A human case of gastric infection by *Pseudoterranova decipiens* larva. Korean J Parasitol..

[7] Shudo R, Yazaki Y, Yamada H, Sugawara K, Takahashi K (2002). Infection with a black *Pseudoterranova decipiens*. Gastrointest Endosc.

[8] Na HK, Seo M, Chai JY, Lee EK, Jeon SM (2013). A case of anisakidosis caused by *Pseudoterranova decipiens* larva. Korean J Parasitol..

[9] Mehrdana F, Bahlool QZ, Skov J, Marana MH, Sindberg D, Mundeling M, et al. Occurrence of zoonotic nematodes *Pseudoterranova decipiens*, *Contracaecum osculatum* and *Anisakis simplex* in cod (*Gadus morhua*) from the Baltic Sea. Vet Parasitol. 2014;205:581–7. 10.1016/j.vetpar.2014.08.027 25224792

[10] Mattiucci S, Nascetti G (2008). Advances and trends in the molecular systematics of anisakid nematodes, with implications for their evolutionary ecology and host-parasite co-evolutionary processes. Adv Parasitol.

[11] Audicana MT, Kennedy MW (2008). *Anisakis simplex*: from obscure infectious worm to inducer of immune hypersensitivity. Clin Microbiol rev.

[12] Dupouy-Camet J, Touabet-Azouzi N, Fréalle E, Van Cauteren D, Yera H, Moneret-Vautrin A (2016). Incidence de l’anisakidose en France. Enquête rétrospective 2010-2014. Bull Epidémiol Hebd.

[13] Dupouy-Camet J, Gay M, Bourgau O, Nouchi A, Leger E, Dei-Cas E (2014). Oesophageal localization: a rare complication of anisakidosis due to *Pseudoterranova*. Presse med.

[14] Pinel C, Beaudevin M, Chermette R, Grillot R, Ambroise-Thomas P (1996). Gastric anisakidosis due to *Pseudoterranova decipiens* larva. Lancet.

[15] Weitzel T, Sugiyama H, Yamasaki H, Ramirez C, Rosas R (1996). (2015) human infections with *Pseudoterranova cattani* nematodes, Chile. Emerg Infect Dis.

[16] Arizono N, Miura T, Yamada M, Tegoshi T, Onishi K (2011). Human infection with *Pseudoterranova azarasi* roundworm. Emerg Infect Dis.

[17] Sohn WM, Seol SY (1994). A human case of gastric anisakiasis by *Pseudoterranova decipiens larva*. Korean J Parasitol.

[18] Zhu X, Gasser RB, Podolska M, Chilton NB (1998). Characterisation of anisakid nematodes with zoonotic potential by nuclear ribosomal DNA sequences. Int J Parasitol.

[19] Martín-Sánchez J, Artacho-Reinoso ME, Díaz-Gavilán M, Valero-López A (2005). Structure of *Anisakis simplex* s.L. populations in a region sympatric for *A. pegreffii* and *A. simplex* s.S. Absence of reproductive isolation between both species. Mol Biochem Parasitol.

[20] Timi JT, Paoletti M, Cimmaruta R, Lanfranchi AL, Alarcos AJ, Garbin L, et al. Molecular identification, morphological characterization and new insights into the ecology of larval *Pseudoterranova cattani* in fishes from the argentine coast with its differentiation from the Antarctic species, *P. decipiens* sp. E (Nematoda: Anisakidae). Vet Parasitol. 2014;199:59–72. 10.1016/j.vetpar.2013.09.033 24161261

[21] Zhu XQ, D’Amelio S, Palm HW, Paggi L, George-Nascimento M, Gasser RB (2002). SSCP-based identification of members within the *Pseudoterranova decipiens* complex (Nematoda: Ascaridoide:Anisakidae) using genetic markers in the internal transcribed spacers of ribosomal DNA. Parasitology.

[22] Mf R, Mascaró C, Fernandez C, Luque F, Sanchez Moreno M, Parras L, et al. Acute intestinal anisakiasis in Spain: a fourth-stage *Anisakis simplex* Larva. Mem Inst Oswaldo Cruz. 1999;94(6):823–6. 10.1590/s0074-02761999000600020 10585662

[23] Pacios E, Arias-Diaz J, Zuloaga J, Gonzalez-Armengol J, Villarroel P, Balibrea JL (2005). Albendazole for the treatment of anisakiasis ileus. Clin Infect Dis.

